# Allogeneic hematopoietic stem cell transplantation for mixed-phenotype acute leukemia: a single-center study

**DOI:** 10.3389/fimmu.2025.1691762

**Published:** 2025-11-18

**Authors:** Mengze Hao, Xiaoli Zhao, Xiaoyu Zhang, Weihua Zhai, Qiaoling Ma, Donglin Yang, Aiming Pang, Sizhou Feng, Yi He, Erlie Jiang, Mingzhe Han

**Affiliations:** 1 State Key Laboratory of Experimental Hematology, National Clinical Research Center for Blood Diseases, Haihe Laboratory of Cell Ecosystem, Institute of Hematology & Blood Diseases Hospital, Chinese Academy of Medical Sciences & Peking Union Medical College, Tianjin, China; 2 Tianjin Institutes of Health Science, Tianjin, China

**Keywords:** mixed-phenotype acute leukemia (MPAL), allogeneic hematopoietic stem cell transplantation (allo-HSCT), prognosis, overall survival (OS), relapse-free survival (RFS), minimal residual disease (MRD)

## Abstract

**Introduction:**

Mixed-phenotype acute leukemia (MPAL) is a rare subtype of acute leukemia with unfavorable outcome. There is no established optimal therapy regime.

**Methods:**

We conducted a retrospective analysis in our transplant center to clarify the efficacy of allogeneic hematopoietic stem cell transplantation (allo-HSCT) in the treatment of MPAL.

**Results:**

This study monitored 61 MPAL patients who underwent allo-HSCT at a single center in China. Haploidentical donor HSCT was 41, matched unrelated donor HSCT was 4, and matched sibling donor HSCT was 16. The median age at diagnosis was 32 years (range, 14-58). The two most common phenotypes were B-lymphoid/myeloid (n=33, 54.1%) and T-lymphoid/myeloid (n=22, 36.1%). In induction treatment, 50 (82.0%) patients received an ALL-like treatment protocol, and 15 of the 17 BCR::ABL1 positive patients received tyrosine kinase inhibitor (TKI) therapy. After induction treatment, 38 (62.3%) patients achieved complete remission (CR). Pre-HSCT 55/61 (90.2%) acquired complete remission (CR) and 46/61 (75.4%) turned minimal residual disease (MRD) -negative. The median follow up time was 28.2 months. The estimated 2-year overall survival (OS) rates after HSCT were 80.0% ± 6.0%. And the relapse-free survival (RFS) probabilities at 2-year were 68.0±7.0%. There was no significant difference in OS and RFS among different types of HSCT. Patients with MRD-positive pre-HSCT was associated with worse OS (P=0.022). Patients who achieved CR after induction therapy had a longer RFS (P=0.033).

**Discussion:**

Allo-HSCT is effective in the treatment of MPAL especially in patients who achieved CR after induction therapy or who got MRD-negative pre-HSCT.

## Introduction

Mixed-phenotype acute leukemia (MPAL) is a rare and heterogeneous disease, accounting for 2% to 5% of all acute leukemia (AL) (1). MPAL shows no clear evidence of differentiation along a single lineage, which is classified based on immunophenotypes into B-lymphoid/myeloid (B-M) and T-lymphoid/myeloid (T-M) subtypes (2). Furthermore, subtypes with specific genetic features, such as MPAL with *BCR::ABL1* fusion or MPAL with *KMT2A*-rearranged, have been defined (2). *BCR::ABL1* fusion is more common in adults, while *KMT2A* rearrangements are more prevalent in pediatric MPAL. These two subgroups account for approximately 19% to 28% of all MPAL cases (3, 4). The prognosis of MPAL is much worse than acute lymphoblastic leukemia (ALL) or acute myeloid leukemia (AML), and the probability of recurrence rate was predicted to be higher in a meta-analysis (5).

The optimal treatment regimen for MPAL is still unclear. Previous reports have shown that induction treatment with an ALL regimen can improve remission rates (5). In the pre-transplant era, the prognosis for MPAL was poor, with a survival rate of approximately 20% (6). Consolidation treatment with allogeneic hematopoietic stem cell transplantation (allo-HSCT) is the preferred approach (6, 7). Previous experiences suggested that a cluster of high-risk genetic features in MPAL was associated with worse outcomes such as *BCR::ABL1* and *KMT2A* rearrangements and some complex karyotypes (3, 4, 8). The addition of a tyrosine kinase inhibitor (TKI) in the subset of *BCR::ABL1*-positive MPAL was well tolerated and recommended (9). A multicenter study by the Acute Leukemia Working Party of the European Society for Blood and Marrow Transplantation (EBMT) recruited 519 *de novo* MPAL patients and reported a 3-year overall survival (OS) rate of 56.3% and a relapse-free survival (RFS) rate of 46.5% for those who underwent HSCT (6). Even with allo-HSCT, OS remains unsatisfactory, and the factors influencing prognosis warrant further investigation. This article aims to analyze the clinical, molecular, and cytogenetic characteristics of a group of 61 MPAL patients, with a particular focus on survival and prognostic factors post-allo-HSCT.

## Materials and methods

### Patients

Sixty-one consecutive patients, aged 14 years or older, with MPAL between August 2008 and December 2023, were recruited in this retrospective study, and all underwent allo-HSCT at the Stem Cell Transplantation Center, Institute of Hematology and Blood Diseases Hospital, Chinese Academy of Medical Sciences. All patients conformed to the definition of MPAL in the 2022 WHO classification of myeloid neoplasms and acute leukemia. All data were collected from clinical records. All cases were diagnosed using standard diagnostic methods, including morphological examination of peripheral blood (PB) and bone marrow (BM) smears, immunophenotyping by flow cytometry (FCM), conventional cytogenetics, and molecular studies. The FCM results at the time of diagnosis were retrospectively reviewed. The study was approved by the Medical Ethics Committee of the Institute of Hematology and Blood Diseases Hospital.

### Treatment protocol for HSCT

All patients were conditioned with a myeloablative conditioning regimen (MAC) with or without total body irradiation (TBI), described as TFAC, TMC, and VBC regimens, and the transplant day was named as day 0. The TFAC conditioning regimen was comprised of TBI (10 Gy, days −9 to −7), fludarabine (30 mg/m^2^/day, days −6 to −4), cytarabine (2 g/m^2^/day, days −6 to −4) or idarubicin (12 mg/m^2^/day, days −6 to −4), and cyclophosphamide (CTX 40 mg/kg/day, days −3 to −2). The TMC conditioning regimen included TBI (10 Gy, days −8 to −6), melphalan (60 mg/m^2^/day, days −5 to −4), and CTX (40 mg/kg/day, days −3 to −2), and the VBC regimen included etoposide (20 mg/kg/day, days −8 to −7), busulfan (3.2 mg/kg/day, days −6 to −4), and CTX (40 mg/kg/day, days −3 to −2). Rabbit antithymocyte globulin (rATG 2.5 mg/kg/day, days −5 to −2) was added to the regimen in the haploidentical stem cell transplantation (HID-HSCT) and matched unrelated donor transplantation (MUD). All recipients received cyclosporine A or tacrolimus and short-term methotrexate as graft-versus-host disease (GVHD) prophylaxis. HID-HSCT recipients received mycophenolate mofetil consistent with our previous experience (10). The stem cell source was peripheral blood stem cells (PBSCs). Immunosuppressive agents can be reduced by 25%–30% at 2 to 3 months post-HSCT and discontinued within 6 months if GVHD has been fully resolved.

### Definitions

Complex karyotypes were defined as the presence of three or more clonal structural chromosomal abnormalities, identified after culturing BM cells for 24 to 48 h in a tissue-culture medium using routine techniques. The date of granulocyte engraftment was the first day of three consecutive days with an absolute neutrophil value greater than 0.5 × 10^9^/L. The time of platelet recovery was defined as the first of seven consecutive days with an absolute platelet count greater than 20 × 10^9^/L in the absence of transfusion. BM aspirate was performed on day 0, day of neutrophil recovery, day 28, day 42, day 60, and day 90 to assess the disease status in the first 3 months after HSCT. Minimal residual disease (MRD) was monitored using FCM for all the patients with a sensitivity of 0.01%. Quantitative real-time polymerase chain reaction (qRT-PCR) was additionally performed only in those who were *BCR::ABL1*-positive (17 cases) or *FLT3-ITD*-positive (7 cases), with a sensitivity of 0.001% (11, 12). Less than 5% blasts in bone marrow were regarded as complete remission (CR), a blast count of more than 20% was defined as no remission (NR), and a blast count of 5%–19% was defined as partial remission (PR). The last follow-up was in November 2024. All patients had a median follow-up of 29.1 months (range 8.8–198.4 months). OS was defined as the time from the infusion to the last follow-up or death. RFS was defined as survival with no hematological relapse. Non-relapse mortality (NRM) was defined as death without previous relapse, and it was calculated by using a cumulative incidence function with death as a competing risk. AGVHD and chronic GVHD (cGVHD) were graded according to international criteria (13, 14).

### Statistical analysis

The probabilities of OS and RFS were assessed using the Kaplan–Meier method, and differences between groups were compared using the log-rank test. The Cox proportional hazards regression model was applied in multivariate analysis to identify independent risk factors, including variables with a *p-*value <0.1, considered statistically significant. The covariates included in the Cox model were the response of induction therapy, MRD pre-HSCT, and conditioning regimen, and the response to induction therapy was treated as a time-dependent covariate. OS and RFS were analyzed using the Cox proportional hazards model. Considering competing risks, the cumulative incidence of relapse (CIR) was calculated by competing risk analysis. Moreover, the incidence of various infections (CMV, EBV, cystitis) as well as of GVHD (including aGVHD and cGVHD) was calculated by cumulative incidence. Appropriately, the chi-square test or Student’s *t*-test was used to compare the distribution of various parameters. Statistical analyses were performed using SPSS 27.0 and R software (version 3.4.3). *P*-values <0.05 were considered as a measure of statistical significance.

## Results

### Patient characteristics

**Figure 1:**
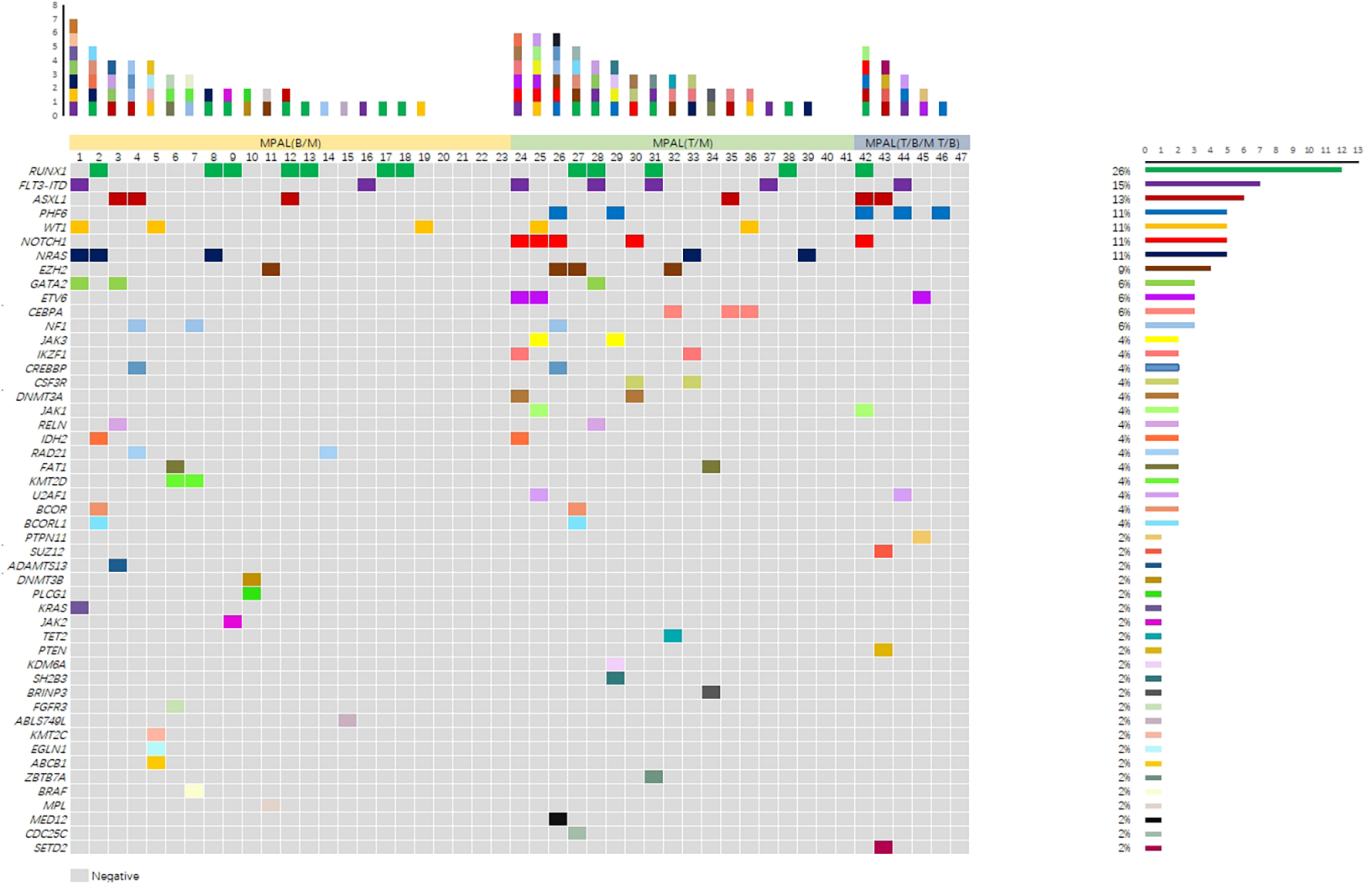
Gene mutations of 47 patients with mixed-phenotype acute leukemia (MPAL).

Among the 61 patients, 33 (54.1%) were diagnosed with myeloid with B-cell marker (B-M) MPAL, 22 (36.1%) with T-lymphoid/myeloid (T-M) MPAL, and 6 (9.8%) with T-lymphoid/B-lymphoid/myeloid (T-B-M) or T-lymphoid/B-lymphoid (T-B) MPAL according to the WHO classification based on immune phenotype. Based on cytogenetics, 17 MPAL patients were *BCR::ABL1*-positive, and 6 patients had *KMT2A* rearrangement, with both phenotypes being B-M. There was a male prevalence (54.1% of patients), and the median age at diagnosis was 32 years (range 14–58). The median percentage of blast in BM was 79%, and the range was 20% to 98.5%. The range of blood cell analysis counts was very large (details in **Table 1**). All patients had hematopoietic cell transplantation-comorbidity index (HCT-CI) scores less than 1 and KPS scores not less than 80. The interval from diagnosis to HSCT ranges from 4 months to 15 months. Cytogenetic analysis was available in 52/61 patients. Karyotype was normal in 15 patients (24.6%) and complex in 12 (17.6%). Other cytogenetic abnormalities [such as +8, +21, +22, 20q−, t (9;22)] were found in 26 patients. Genetic data were available for 47/61 patients, with 7 patients having no gene mutations detected. More detailed information on gene mutations is shown in **Figure 1**. The most frequent gene mutations were *RUNX1* (12 cases), *FLT3-ITD* (7 cases), *ASXL1* (6 cases), *PHF6* (5 cases), *WT1* (5 cases), *NOTCH1* (5 cases), and *NRAS* (5 cases). Detailed clinical characteristics are presented in **Table 1**.

**Table 1:** Characteristics of the 61 MPAL patients.

Characteristics	Patients, *N* (%), *N* = 61
Gender (male/female)	33/28 (54.1%/45.9%/)
Median age at diagnosis (years)	32 (14–58)
Median WBC count (×10^9^/L)	23.5 (0.4–527.3)
Median HGB count (g/L)	89 (46–298)
Median PLT count (×10^9^/L)	77 (9–403)
Myeloblast in BM	79% (20%–98.5%)
KPS scores (≥80)	61 (100%)
HCT-CI scores	
0	50 (82.0%)
1	11 (18.0%)
Time from diagnosis to HSCT	
4–6 months	31 (50.8%)
7–15 months	30 (49.2%)
Splenomegaly	
None	19 (31.1%)
Mild/moderate	21 (34.4%)
Severe	4 (6.6%)
NA	17 (27.9%)
Cytogenetics/molecular type at diagnosis	
Normal karyotype	14 (23.0%)
Abnormal karyotype	26 (42.6%)
t(9;22)	10
+8	2
20q−	1
+22	1
Others	12
Complex karyotype	12 (19.7%)
NA	9 (14.8%)
CNSL	4 (6.6%)
Type of MPAL by WHO(Cytogenetics)	
BCR::ABL1-positive	17 (27.9%)
MLL rearrangement	6 (9.8%)
Others	38 (62.3%)
Type of MPAL by the WHO (immune phenotype)	
B-M	33 (54.1%)
T-M	22 (36.1%)
T-B-M/T-B	6 (9.8%)
MRD pre-HSCT	
Negative	46 (75.4%)
Positive	15 (24.6%)
Conditioning regimen	
With TBI	49 (80.3%)
Without TBI	12 (19.7%)
Donor type	
MSD	16 (26.2%)
MUD/HID	4 + 41 (73.8%)

MPAL, mixed-phenotype acute leukemia; BM, bone marrow; HCT-CI, hematopoietic cell transplantation-comorbidity index; allo-HSCT, allogeneic hematopoietic stem cell transplantation; CNSL, central nervous system leukemia; B-M, B-lymphoid/myeloid, T-M: T-lymphoid/myeloid; T-B-M, T-lymphoid/B-lymphoid/myeloid; T-B, T-lymphoid/B-lymphoid; TBI, total body irradiation; MSD, matched sibling donor; MUD, matched unrelated donor; HID, haploidentical donor.

### Chemotherapy and response

Fifty of the 61 patients (82%) received an ALL-like induction therapy based on the vindesine and prednisone (VP) regimen, 6 patients (9.8%) were treated using an AML-like induction based on cytarabine and daunorubicin/idarubicin (DA/IA), and the remaining 5 patients (8.2%) received both ALL-like and AML-like treatments. The CR rates after induction therapy of the three groups were 68.0% (34/50), 16.7% (1/6), and 60.0% (3/5), respectively (**Supplementary File 1**). Furthermore, we provided a detailed description of the evaluation of MRD after induction therapy by using FCM and qRT-PCR methods in **Supplementary File 1**. In total, 38 of 61 (62.3%) patients achieved a CR after induction, 6 (9.8%) PR, and 17 (27.9%) NR. Patients who received ALL-like therapy had higher CR rates after induction. However, there was no significant difference in statistics (*P* = 0.084%; OR = 0.269; 95% CI 0.069, 1.053). We recruited 17 *BCR::ABL1-*positive MPAL patients; 15 received TKI combined with induction chemotherapy: 8 with imatinib, 4 with dasatinib, 2 with orebatinib, and 1 with flumatinib. As a result, 12 cases had CR and 5 had NR after induction treatment. Two patients did not receive TKI during induction. One patient received both ALL-like and AML-like regimens for induction without TKI and had a CR. Consolidation chemotherapy was combined with TKI. Another patient received three cycles of AML-like induction without TKI but still achieved NR. In the fourth cycle, the patient received re-induction with TKI and achieved CR.

We conducted a univariate analysis on factors affecting response after induction chemotherapy, including a series of variables such as age (cutoff at 30 years), gender, induction treatment, gene mutation, karyotype (complex vs. others), and types of MPAL according to the WHO (immune phenotype). Patients with no gene mutation had a higher CR rate after induction therapy (7/7 vs. 22/40, *P* = 0.024). After induction, all 17 NR cases received the ALL-like re-induction therapy, with 13 achieving a CR and 8 cases (61.5%) being MRD-negative. Before allo-HSCT, 55/61 (90.2%) acquired a CR and 46/61 (75.4%) turned MRD-negative.

### Allo-HSCT and survival

In the entire cohort, all 61 MPAL patients underwent allo-HSCT using PBSCs as the stem cell sources. Of these, 41 (67.2%) had HID-HSCT, 4 (6.6%) had MUD-HSCT, and 16 (26.2%) had matched sibling donor (MSD)-HSCT. All of them received myeloablative conditioning, with 49 patients (80.3%) receiving regimens that included TBI. The MRD of the infusion day was negative in 53 patients and positive in 8. The infusion number of mononuclear cells (MNCs) was 10.3 (4.29–18.78) × 10^8^/kg, and the number of CD34-positive cells was 3.43 (1.93–11.64) × 10^6^/kg. The median times to neutrophil and platelet engraftment were 13 (range 10–21) days and 14 (range 10–180) days, respectively.

**Figure 2:**
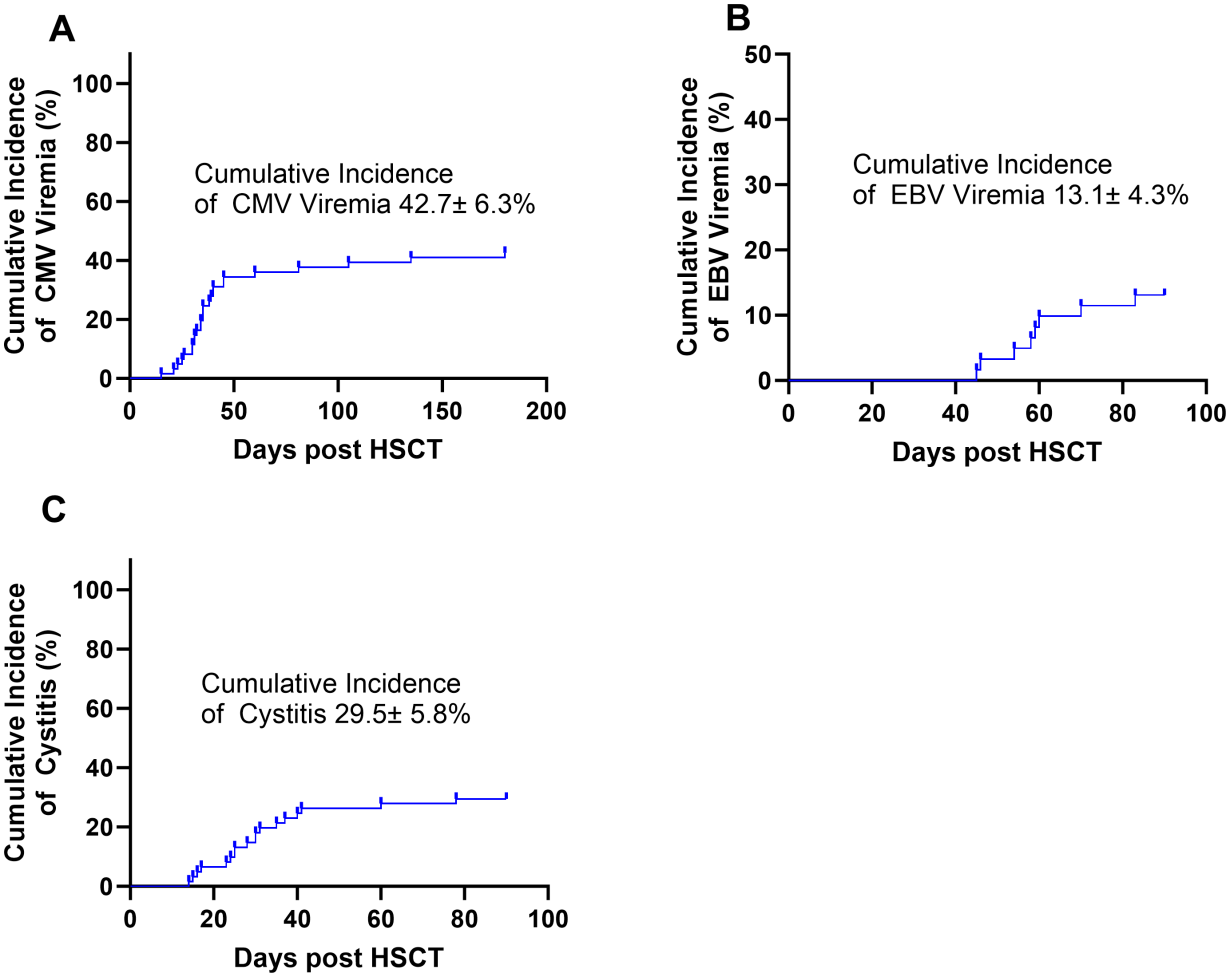
**(A)** The cumulative incidence of CMV reactivation was 42.7% ± 6.3% at 180 days. **(B)** The cumulative incidence of EBV reactivation was 13.1% ± 4.3% at 90 days. **(C)** The cumulative incidence of cystitis was 29.5% ± 5.8% at 90 days.

**Figure 3:**
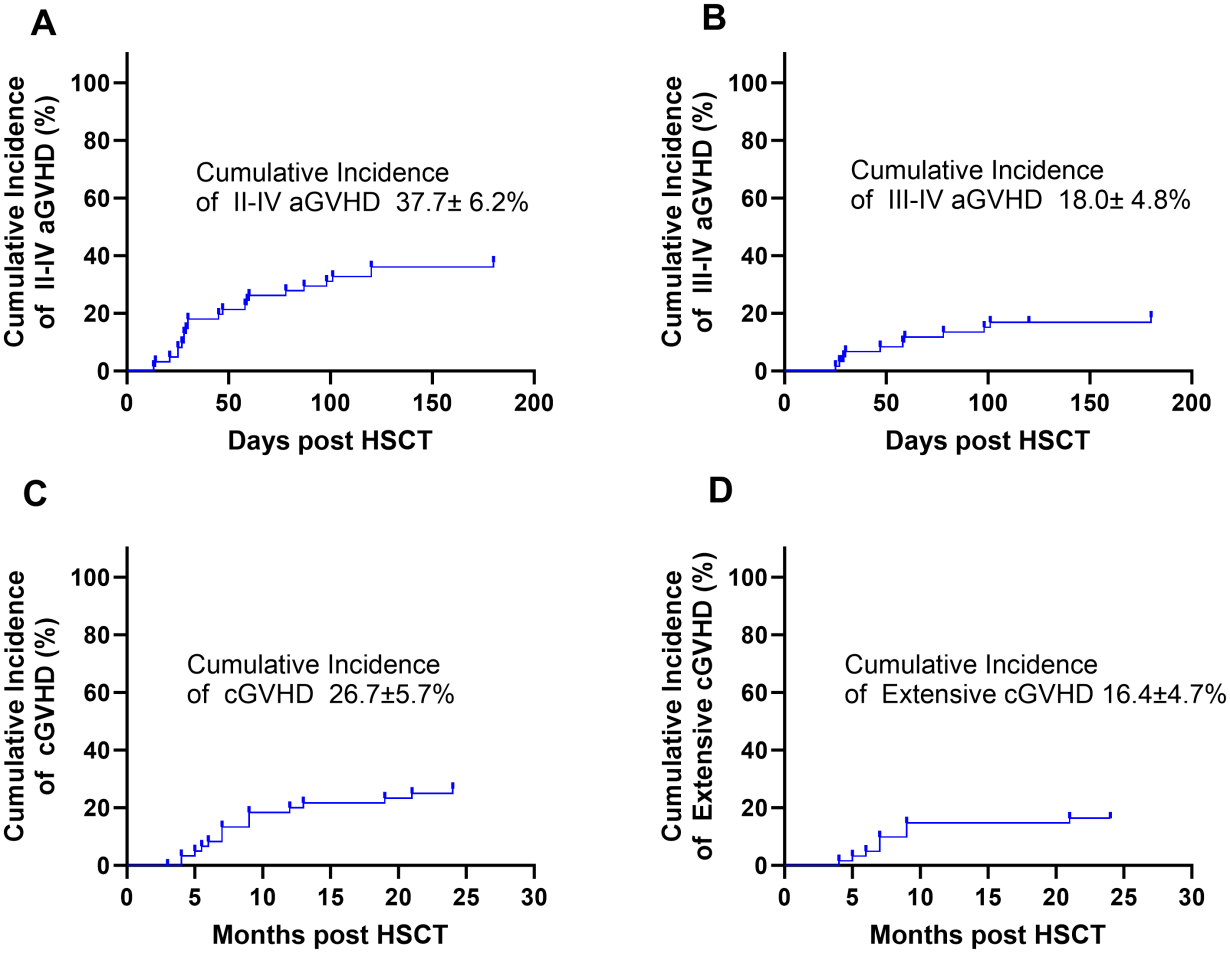
**(A)** The cumulative incidence of grade II–IV acute graft-versus-host disease (aGVHD) at 180 days was 37.7% ± 6.2%. **(B)** The cumulative incidence of grade III–IV aGVHD at 180 days was 18.0% ± 4.8%. **(C)** The cumulative incidence of chronic graft-versus-host disease (cGVHD) at 24 months was 26.7% ± 5.7%. **(D)** The cumulative incidence of extensive cGVHD at 24 months was 16.4% ± 4.7%.

Post-HSCT complications included the following: the cumulative incidence of CMV reactivation at 180 days was 42.7% ± 6.3% (**Figure 2A**), the cumulative incidence of EBV reactivation at 90 days was 13.1% ± 4.3% (**Figure 2B**), and the cumulative incidence of cystitis at 90 days was 29.5% ± 5.8% (**Figure 2C**). Grade II–IV aGVHD cumulative incidence at 180 days was 37.7% ± 6.2% (**Figure 3A**), and grade III–IV aGVHD cumulative incidence at 180 days was 18.0% ± 4.8% (**Figure 3B**). cGVHD cumulative incidence at 24 months was 26.7% ± 5.7% (**Figure 3C**), and extensive cGVHD cumulative incidence at 24 months was 16.4% ± 4.7% (**Figure 3D**).

In the 61 MPAL patients, 9 cases were MRD-positive and 6 cases achieved NR. During the infusion day, 7 out of 9 became MRD-negative, and in 6 patients who achieved NR, 1 turned MRD-negative; however, the other 5 still achieved NR on day 0. The specific details are added in **Supplementary File 2**. Twenty-five (41.0%) patients received maintenance therapy after 2 to 3 months post-allo-HSCT with no aGVHD, no infection, and no stable blood count, including 11 with TKI, 11 with Venclexta, 1 with azacitidine, and 2 with chidamide. Of the 11 patients who were treated with TKIs, 3 patients received orebatinib, 7 received imatinib, and 1 received dasatinib. Only one patient was unable to tolerate imatinib due to nausea and vomiting, hence was switched to fumatinib for maintenance treatment. The remaining 24 patients were able to tolerate the maintenance therapy drugs, with a treatment duration ranging from 1.5 months to 30 months (**Supplementary File 3**). However, there were no statistically significant differences in the cumulative incidence of relapse or NRM between patients who received maintenance therapy and those who did not (**Supplementary File 4A, B**).

After HSCT, 17 (17/61, 27.9%) patients relapsed, and 12 patients accepted donor lymphocyte infusions (DLIs). Six patients became MRD-negative after DLI, while the other six still achieved NR. The specific details are added in **Supplementary File 5**. At the latest follow-up, 50 patients (82.0%) were alive, with 43 of these patients being in CR. Eleven patients (18.0%) have died, with the majority (10 patients, 90.9%) succumbing to relapse, and only one dying from infections.

**Figure 4:**
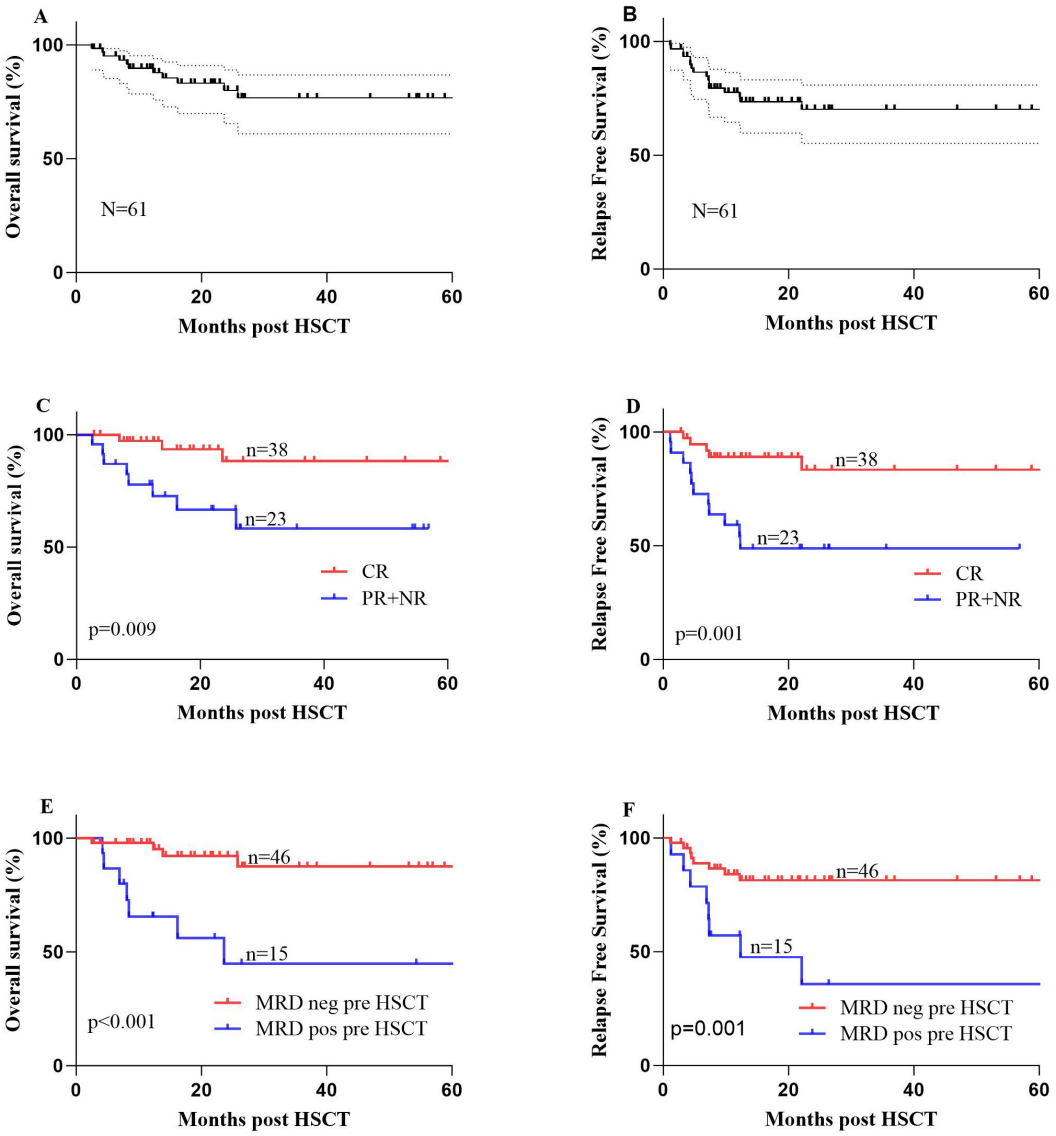
**(A)** The 1- and 2-year overall survival (OS) rates were 89.0% ± 4.0% and 80.0% ± 6.0%, respectively, for 61 patients with mixed-phenotype acute leukemia (MAPL) undergoing allogeneic hematopoietic stem cell transplantation (allo-HSCT). **(B)** The 1- and 2-year relapse-free survival (RFS) rates were 75.0% ± 6.0% and 68% ± 7.0%, respectively, for 61 patients with MAPL undergoing allo-HSCT. **(C)** OS of the two groups of patients with MPAL undergoing allo-HSCT: patients who achieved complete remission (CR) after induction therapy and patients who achieved partial remission (PR) or no remission (NR) after induction therapy. **(D)** RFS of the two groups of patients with MPAL undergoing allo-HSCT: patients who achieved CR after induction therapy and patients who achieved PR or NR after induction therapy. **(E)** OS of the two groups of patients with MPAL undergoing allo-HSCT: patients who were minimal residual disease (MRD)-negative before allo-HSCT and patients who were MRD-positive before allo-HSCT. **(F)** RFS of the two groups of patients with MPAL undergoing allo-HSCT: patients who were MRD-negative before allo-HSCT and patients who were MRD-positive before allo-HSCT.

The estimated 1-, 2-, and 5-year OS rates after HSCT were 89.0% ± 4.0%, 80.0% ± 6.0%, and 76.0% ± 7.0%, respectively. The RFS rates at 1, 2, and 5 years were 75.0% ± 6.0%, 68% ± 7.0%, and 68% ± 7.0%, respectively. **Table 2** presents the specific prognostic data. The 3-year confidence interval (CI) for NRM was 2.3% ± 0.05% for the entire cohort (**Supplementary File 4C**). The 1- and 3-year cumulative recurrence rates were 23.7% ± 0.31% and 31.0% ± 0.43%, respectively (**Supplementary File 4C**). Furthermore, the survival curves for OS and RFS are depicted in **Figures 4A, B**.

**Table 2:** Univariate analysis of the OS and RFS of MPAL patients with allo-HSCT.

Factor	*N*	1-year OS (%)	2-year OS (%)	*P*-value	1-year RFS (%)	2-year RFS (%)	*P*-value
Age/years				0.209			***0.005***
≤30	25	83.8 ± 7.4	69.8 ± 9.7		60.0 ± 9.8	50.1 ± 10.4	
>30	36	93.9 ± 4.2	89.2 ± 6.1		88.1 ± 5.6	84.4 ± 6.5	
Gender				0.218			0.174
Male	33	84.3 ± 6.5	71.4 ± 8.9		72.7 ± 7.8	60.0 ± 9.4	
Female	28	96.2 ± 3.8	91.6 ± 5.7		80.7 ± 7.8	80.7 ± 7.8	
Chromosome karyotype				0.184			0.314
No complex karyotype	41	94.8 ± 3.6	82.5 ± 7.4		79.3 ± 6.6	70.7 ± 8.4	
Complex karyotype	12	66.7 ± 13.6	66.7 ± 13.6		58.3 ± 14.2	58.3 ± 14.2	
NA	8	–	87.5 ± 11.7		87.5 ± 11.7	75.0 ± 15.3	
Type of ALAL/MPAL by the WHO (cytogenetics)				0.317			0.363
BCR::ABL1-positive	17	93.3 ± 6.4	77.5 ± 11.6		68.2 ± 11.8	52.6 ± 13.4	
MLL rearrangement	6	66.7 ± 19.2	66.7 ± 19.2		66.7 ± 19.2	66.7 ± 19.2	
Others	38	91.9 ± 4.5	84.8 ± 6.4		81.2 ± 6.4	77.8 ± 7.0	
Type of ALAL/MPAL by the WHO (immune phenotype)				0.284			0.323
My+B	33	90.1 ± 5.4	77.4 ± 8.3		77.6 ± 7.5	68.6 ± 9.0	
My+T	22	95.5 ± 4.4	89.1 ± 7.4		81.8 ± 8.2	75.5 ± 9.7	
My+T+B/T+B	6	66.7 ± 19.2	66.7 ± 19.2		50.0 ± 20.4	50.0 ± 20.4	
Type of 2008 WHO				0.433			0.694
Biphenotype	29	89.5 ± 5.7	84.3 ± 7.4		79.3 ± 7.5	64.0 ± 10.3	
Bilineal	32	89.9 ± 5.5	76.1 ± 8.9		73.0 ± 8.2	73.0 ± 8.2	
Induction therapy				***0.009***			***0.001***
CR	38	97.2 ± 2.7	88.3 ± 6.6		89.0 ± 5.2	83.4 ± 7.3	
PR+NR	6 + 17	77.8 ± 8.8	66.6 ± 10.5		56.5 ± 10.3	46.6 ± 10.6	
MRD pre-HSCT				***<0.001***			***0.001***
Negative	46	97.8 ± 2.2	92.1 ± 4.4		84.0 ± 5.5	81.3 ± 6.0	
Positive	15	65.5 ± 12.6	44.9 ± 14.9		53.3 ± 12.9	33.3 ± 13.9	
Conditioning regimen				0.210			0.116
With TBI	49	91.4 ± 4.1	81.8 ± 6.5		78.9 ± 5.9	76.3 ± 6.3	
Without TBI	12	83.3 ± 10.8	72.9 ± 13.5		66.7 ± 13.6	38.9 ± 18.5	
Donor type				0.715			0.383
MSD	16	93.8 ± 6.1	79.1 ± 10.8		61.9 ± 12.3	61.9 ± 12.3	
MUD/HID	4 + 41	88.2 ± 5.0	81.6 ± 6.4		81.7 ± 5.9	70.9 ± 7.9	
ABO type match				0.711			0.224
Yes	36	91.2 ± 4.9	79.1 ± 7.9		82.9 ± 6.4	74.5 ± 8.1	
No	25	87.7 ± 6.7	81.4 ± 8.7		66.5 ± 9.7	61.4 ± 10.2	
Gender match				0.886			0.319
F to M	12	90.9 ± 8.7	79.5 ± 13.1		83.3 ± 10.8	83.3 ± 10.8	
Others	49	89.4 ± 4.5	79.9 ± 6.6		74.6 ± 6.4	65.0 ± 7.7	
MRD 0 days				***0.002***			***<0.001***
Negative	53	96.1 ± 2.7	85.1 ± 5.8		84.2 ± 5.1	75.9 ± 6.6	
Positive	8	50.0 ± 17.7	50.0 ± 17.7		25.0 ± 15.3	25.0 ± 15.3	
CMV reactivation				0.853			0.697
Yes	26	88.5 ± 6.3	80.0 ± 8.0		76.9 ± 8.3	72.9 ± 8.8	
No	35	90.8 ± 5.1	78.9 ± 9.2		75.6 ± 7.6	63.4 ± 10.5	
EBV reactivation				0.235			0.111
Yes	8	–	–		–	–	
No	53	88.4 ± 4.4	77.7 ± 6.4		73.4 ± 6.1	65.1 ± 7.1	
Cystitis				0.566			0.622
Yes	18	82.2 ± 9.3	75.9 ± 10.6		71.1 ± 10.9	63.2 ± 12.2	
No	43	92.8 ± 4.0	81.8 ± 7.0		78.3 ± 6.4	72.1 ± 7.2	
aGVHD				0.063			***0.036***
No/I grade	38	91.4 ± 4.7	88.0 ± 5.6		83.5 ± 6.2	78.3 ± 7.7	
Yes II–IV grade	23	86.7 ± 7.1	66.8 ± 11.6		64.2 ± 10.2	51.4 ± 11.5	
cGVHD				0.972			0.916
Yes	16	–	91.7 ± 8.0		87.5 ± 8.3	54.7 ± 18.0	
No	45	85.8 ± 5.4	82.9 ± 5.9		72.0 ± 6.9	72.0 ± 6.9	
Maintenance therapy				0.096			0.211
Yes	25	–	91.7 ± 8.0		83.6 ± 7.5	74.3 ± 11.0	
No	36	82.5 ± 6.5	71.8 ± 8.1		71.0 ± 7.7	64.3 ± 8.4	

MPAL, mixed-phenotype acute leukemia; OS, overall survival; RFS, relapse-free survival; CR, complete remission; PR, partial remission; NR, no remission; allo-HSCT, allogeneic hematopoietic stem cell transplantation; MRD, minimal residual disease; TBI, total body irradiation; MSD, matched sibling donor; MUD, matched unrelated donor; HID, haploidentical donor; F, female; M, male; GVHD, graft-versus-host disease; aGVHD, acute graft-versus-host disease; cGVHD, chronic graft-versus-host disease. Bold values, statistically significant.

### Prognosticators for MPAL with allo-HSCT

Among the MPAL patients, we conducted univariate analyses for OS and RFS in the entire group, considering the following variables separately: age (with a cutoff at 30 years), gender, types of MPAL according to various classification systems, chromosome karyotype, status after induction therapy, MRD status pre-HSCT, conditioning regimen, donor type, gender match (F to M vs. others), ABO type match, allo-HSCT-related complications, and maintenance treatment. Consequently, patients who achieved CR after induction therapy had significantly longer OS (88.3% ± 6.6% vs. 66.6% ± 10.5%, *P* = 0.009) and better RFS (83.4% ± 7.3% vs. 46.6% ± 10.6%, *P* = 0.001) (**Figures 4C, D**). Additionally, the MRD status pre-HSCT and on the infusion day both played a significant role in both OS and RFS survival (negative vs. positive, *P* < 0.05) (**Figures 4E, F**). Recurrence affects the prognosis of OS, resulting in dismal survival rates (44.3% ± 12.4% vs. 96.7% ± 3.3%, *P* < 0.001).

In the multivariate analysis, a favorable variable for OS was achieving MRD negativity before allo-HSCT (*P* = 0.022, HR 0.22, 95% CI 0.06–0.80) (**Table 3**). According to RFS, CR after induction (*P* = 0.033, HR 0.30, 95% CI 0.10–0.91) was an independent protective factor (**Table 4**).

**Table 3:** Multivariate analysis of the OS of MPAL patients with allo-HSCT.

Factor	*N* = 61 (%)	Multivariate analysis			
		*β*	Wald	HR 95% CI	*P*-value
Induction therapy					0.105
PR+NR	23 (37.7%)	0		1.00	
CR	38 (62.3%)	−1.15	2.63	0.32 (0.08–1.27)	
MRD pre-HSCT					***0.022***
Positive	15 (24.6%)	0		1.00	
Negative	46 (75.4%)	−1.52	5.28	0.22 (0.06–0.80)	
Conditioning regimen					0.365
Without TBI	12 (19.7%)	0			
With TBI	49 (80.3%)	−0.58	0.82	0.56 (0.16–1.97)	

MPAL, mixed-phenotype acute leukemia; allo-HSCT, allogeneic hematopoietic stem cell transplantation; OS, overall survival; CR, complete remission; PR, partial remission; NR, no remission; MRD, minimal residual disease; TBI, total body irradiation. Bold values, statistically significant.

**Table 4:** Multivariate analysis of the RFS of MPAL patients with allo-HSCT.

Factor	*N* = 61 (%)	Multivariate analysis			
		*β*	Wald	HR 95% CI	*P*-value
Induction therapy					***0.033***
PR+NR	23 (37.7%)	0		1.00	
CR	38 (62.3%)	−1.22	4.55	0.30 (0.10–0.91)	
MRD pre-HSCT					0.089
Positive	15 (24.6%)	0		1.00	
Negative	46 (75.4%)	−0.91	2.90	0.40 (0.14–1.15)	
Conditioning regimen					0.442
Without TBI	12 (19.7%)	0			
With TBI	49 (80.3%)	−0.41	0.59	0.67 (0.24–1.88)	

MPAL, mixed-phenotype acute leukemia; allo-HSCT, allogeneic hematopoietic stem cell transplantation; RFS, relapse-free survival; CR, complete remission; PR, partial remission; NR, no remission; MRD, minimal residual disease; TBI, total body irradiation. Bold values, statistically significant.

## Discussion

MPAL is a rare type of AL, characterized by the simultaneous display of more than one lineage, such as myeloid and B-lymphoid or T-lymphoid in MPAL (15–17). In addition to *BCR::ABL1* and *KMT2A*-rearranged cases, *ZNF384* and *BCL11B* rearrangements, as novel genetic findings, have been added as subtypes (15). We found that B-M and T-M MPAL are the most common types, consistent with earlier reports (5, 18). With the advancement of next-generation targeted sequencing and molecular biology technologies, our understanding of the gene mutation spectrum in MPAL patients has deepened. In MPAL, mutations are primarily found in genes related to epigenetic modification, signaling pathways, and transcription factors, and they vary depending on the patients’ ages. Pediatric MPAL primarily exhibits mutations in genes such as *ZNF384*, *WT1*, and *CEBPA*, while adults mainly have mutations in *RUNX1*, *NOTCH1*, and *DNMT3A* (19). In our data, no patient was younger than 14 years, and *RUNX1*, occurring in 12 cases, was the gene with the highest frequency. This mutation was considered a poor prognostic indicator in AL. Compared to mutations in ALL and AML, B-M MPAL had a similar expression profile to B-ALL, and T-M MPAL had a similar expression profile to ETP-ALL, both mainly featuring mutations in genes related to *WT1*, *RAS*, and the *JAK-STAT* pathway (19, 20). Neither *PHF6* nor *NOTCH1* mutations were found in B-M MPAL, but they were enriched in T-M MPAL. It has been reported that *PHF6* and *NOTCH1* mutations were often implicated in the development of T-ALL and less frequently in AML and other myeloid neoplasms (21). All of the above indicate the genetic differences among subgroups of MPAL. Extensive and in-depth exploration of the genomics of MPAL highlights its complexity, and this might lead to improvements in classification and nomenclature in the future.

There are still no confirmed therapeutic guidelines for MPAL. Until now, only a series of small retrospective studies have been conducted. A meta-analysis found that ALL regimens were more likely to achieve CRs after induction than AML regimens and that AML induction had poorer efficacy in multivariable analysis (5, 22). Another study with 49 MPAL patients showed similar survival rates despite different induction therapy types (23). Our data show that patients who received the ALL regimen achieved a higher CR rate; however, the *P*-values did not show statistical significance. In the pre-transplant era, the prognosis of MPAL patients remains poor, although remission could be achieved by chemotherapy. Research revealed that patients who received allo-HSCT lived longer, with a 2-year survival rate of 57.8%, as compared to 20.2% among patients who did not receive HSCT (23). In 2024, the authors renewed their data showing that HSCT patients had better progression-free survival (PFS) (*P* = 0.025) and OS (*P* = 0.011) compared to those not transplanted (24), especially those who achieved CR after induction therapy (24). In our cohort, all the patients underwent allo-HSCT, with 1- and 2-year OS rates of 89.0% ± 4.0% and 80.0% ± 6.0%, respectively. The survival rates were consistent with recent research findings (24), even better than prior literature (16). These better outcomes likely stem from our cohort’s younger median age of 32 years (no patient was older than 60) compared to the previous report’s median age of 49 years (range 18–62) (16). Moreover, the MRD-negative rate achieved by our patients before transplantation was higher (75.4% vs. 48.0%) (16).

In addition, we assessed the prognostic factors for MPAL patients after allo-HSCT. Several predictors with better OS were identified, such as achieving CR after induction therapy and being MRD-negative pre-HSCT or on the day of infusion. Moreover, MRD-negative pre-HSCT was an independent predictor for OS of MPAL patients. This is similar to a previous study, where patients who were MRD-negative pre-HSCT had superior survival post-HSCT of AML and ALL (16, 25, 26). However, there were no statistically significant differences in HSCT types—whether HID-HSCT, MUD-HSCT, or MSD-HSCT—making any of these a feasible strategy for MPAL patients.

However, relapse remains the major cause of treatment failure in AL patients who undergo allo-HSCT. Of the 61 patients, 17 had recurrence, 10 of whom died from relapse. These patients might benefit from post-transplantation maintenance therapy, especially high-risk AL patients, who are usually maintained for 1 or 2 years after allo-HSCT, particularly in the absence of GVHD (27, 28). In our study, 25 out of 61 patients received maintenance therapy after HSCT, including venetoclax, azacitidine, and TKIs (for *BCR::ABL1-*positive MPAL), with treatment durations ranging from 1.5 months to 1–2 years. Patients who received maintenance therapy seemed to have better survival (2-year survival rates: 91.7% ± 8.0% vs.71.8% ± 8.1%, *P* = 0.096) and a lower recurrence rate [5 in 25 (20.0%) vs. 12 in 36 (33.3%)].

As a single-center retrospective study, there are obvious limitations. In summary, MPAL is a heterogeneous lethal disease with no standardized guidelines for diagnosis and treatment. An ALL-like regimen should be considered for use in induction therapy. Allo-HSCT, whether HID-HSCT, MUD-HSCT, or MSD-HSCT, is a feasible strategy for MPAL patients, especially those who have achieved CR after induction therapy and those who are MRD-negative pre-HSCT. More prospective, multicenter, large-scale studies are still needed in the near future (29).

## Data Availability

The data analyzed in this study is subject to the following licenses/restrictions: Due to the nature of this research, participants of this study did not agree for their data to be shared publicly, so supporting data is not available. Requests to access these datasets should be directed to haomengze@ihcams.ac.cn.
